# Superficial cervical plexus blockade improves pain control after thyroidectomy: A randomized controlled trial

**DOI:** 10.6061/clinics/2019/e605

**Published:** 2019-09-10

**Authors:** Taís Fonseca Goulart, Vergilius José Furtado de Araujo-Filho, Claudio Roberto Cernea, Leandro Luongo Matos

**Affiliations:** I Departamento de Anestesia, Hospital das Clinicas HCFMUSP, Faculdade de Medicina, Universidade de Sao Paulo, Sao Paulo, SP, BR; II Departamento de Cirurgia de Cabeca e Pescoco, Hospital das Clinicas HCFMUSP, Faculdade de Medicina, Universidade de Sao Paulo, Sao Paulo, SP, BR; III Departamento de Cirurgia de Cabeca e Pescoco, Instituto do Cancer do Estado de Sao Paulo (ICESP), Hospital das Clinicas HCFMUSP, Faculdade de Medicina, Universidade de Sao Paulo, Sao Paulo, SP, BR

**Keywords:** Thyroidectomy, Analgesia, Cervical Plexus Block, Pain, Thyroid Neoplasm

## Abstract

**OJECTIVES:**

The aim was to evaluate the ability of bilateral superficial cervical plexus blockade to control pain and to reduce the side effects of general anesthesia in patients submitted to thyroidectomy.

**METHODS:**

In this randomized controlled trial, we prospectively studied 100 consecutive patients who underwent total thyroidectomy. The simple random patient sample was divided into two groups: 50 patients received general anesthesia alone (group 1 [G1]), and 50 patients received general anesthesia with bilateral superficial cervical plexus blockade (group 2 [G2]). Statistical analyses were performed, and a 5% significance level was adopted.

**RESULTS:**

The mean arterial blood pressure and heart rate were 12% lower in G2 patients than in G1 patients 60 minutes after surgery (101 mmHg for G1 *vs.* 92.3 mmHg for G2; *p*<0.001). G2 patients reported less pain than G1 patients, and opioid consumption was lower in G2 patients than in G1 patients, not upon postanesthesia care unit arrival, but at 30 minutes (2% *vs.* 34%; *p*<0.001, respectively), 45 minutes (0% *vs.* 16%; *p*=0.006, respectively), and 4 hours postoperatively (6% *vs.* 20%; *p*=0.037, respectively). The incidence of nausea and vomiting was lower in G2 patients than in G1 patients from 45 minutes (0% *vs.* 16%; *p*=0.006, respectively) to 8 hours postoperatively (0% *vs.* 14%; *p*=0.012, respectively).

**CONCLUSIONS:**

The present study demonstrated that the combination of bilateral superficial cervical plexus blockade with general anesthesia for thyroidectomy is feasible, safe, and effective for achieving pain control and improving patient outcomes.

## INTRODUCTION

Pain control is a necessary aspect of care during the postoperative period. Pain is considered the fifth vital sign, and its control reduces clinical and surgical complications, reduces the incidence of side effects, and improves patient outcomes 1.

Management of the postoperative period after thyroidectomy, which extends from extubation to hospital discharge, is crucial for the success of the procedure. Arterial blood pressure control and the incidence of postoperative nausea and vomiting (PONV) are related to reduced postoperative bleeding and cervical hematoma. However, uncontrolled pain can increase postoperative bleeding and cervical hematoma via the adrenergic pathway and catecholamine secretion. Furthermore, the concomitant use of analgesics, such as opioids, which have side effects that include nausea and vomiting, may worsen a patient's clinical condition 2-4.

The superficial cervical plexus innervates the anterior cervical skin through the primary rami of C2-C4. For thyroidectomy, the effectiveness of bilateral superficial cervical plexus blockade (BSCPB) in combination with general anesthesia remains controversial with respect to pain control and a reduction in the side effects of postoperative general anesthesia 5. The aim of this randomized controlled trial was to evaluate the influence of BSCPB on pain control and on the ability to reduce the side effects of general anesthesia in patients submitted to thyroidectomy.

## METHODS

This randomized controlled trial was approved by the Institutional Research Ethics Board. After obtaining informed consent, we prospectively studied a series of 100 consecutive patients who underwent total thyroidectomy at a tertiary hospital over a period of 2 years.

The inclusion criteria were as follows: patients with American Society of Anesthesiologists (ASA) physical status I or II, aged between 25 and 65 years old, and with a body mass index (BMI) between 20 and 30. The exclusion criteria were as follows: patients with ASA physical status III or IV, patients with rheumatic or neurological diseases (peripheral or central neuropathies), and patients being treated for chronic pain because of the long-term use of corticosteroids, opioids, or other pain medications that may affect the results.

The simple random patient sample was divided into two groups: 50 patients received total intravenous general anesthesia (TIVA) (group 1 [G1]), and 50 patients received TIVA in combination with BSCPB (group 2 [G2]). Patients were informed in advance that they would be assigned to either the TIVA group or the TIVA plus BSCPB group.

All anesthetic procedures were performed by the same anesthesiologist in both groups; all surgical procedures were performed by the same head and neck surgeon V.J.F.A.F.

### Evaluation Before Anesthesia

Both groups were evaluated by the same anesthesiologist before anesthesia. Anesthesia was induced by administering 0.75 mg/kg intramuscular midazolam 30 minutes before the surgery and prior to entering the operating room.

### Anesthesia in the Operating Room (OR)

The following procedures were adopted at the OR:

1. Monitoring of the G1 and G2 patients was performed using continuous cardioscopy, pulse oximetry, noninvasive arterial blood pressure (NIABP), heart rate (HR), continuous capnography, temperature determined by an esophageal thermometer, and bispectral index (BIS). The data were recorded at three time points: M1, before anesthesia induction; M2, just after induction and oral intubation; and M3, just after extubation.
2. The blood glucose levels of the G1 and G2 patients were maintained between 100 and 150 mg/dl and evaluated at M1 and M3.
3. Anesthesia induction in G1 patients was performed as follows: after 20-gauge peripheral venous catheterization and preoxygenation for 5 minutes via a face mask at an oxygen inspired fraction (FiO_2_) of 100%, anesthesia was slowly induced by an intravenous bolus of fentanyl at 2 mcg/kg, propofol at 2.5 mg/kg, and a bolus of cisatracurium at 0.1 mg/kg. After 10 minutes of facial mask ventilation and a BIS under 40, tracheal intubation was performed. The mechanical ventilation parameters were a tidal volume of 8 ml/kg, positive end-expiratory pressure (PEEP) of 5 mmHg, respiratory frequency to maintain capnography between 30 and 35 mmHg, and FiO_2_ of 50% (oxygen and air). The anesthesia was maintained by the continuous infusion of propofol at an average dose of 0.05 mg/kg/minute and remifentanil at 0.5 mcg/kg/minute (to maintain a BIS under 60 and the mean arterial blood pressure at 10% to 20% below the basal awake levels). Neither additional doses of fentanyl nor cisatracurium were administered. For intraoperative hydration, Ringer's lactate solution was infused at 5 ml/kg/hour.
4. Anesthesia induction in G2 patients was performed as follows: after anesthesia induction as described above for G1 patients and before the beginning of surgery, the patients' heads were rotated to the contralateral side of the blockade. After antisepsis, the posterior border of the clavicular sternocleidomastoid head muscle (inserts from the mastoid process to the clavicle) was identified. The puncture was made at the midpoint of a line drawn from the mastoid to the C6 transverse process (Chassaignac's tubercle), which may coincide with the junction between the external jugular vein and the muscle; here, a 22-gauge needle was inserted. After aspiration and no evidence of blood, 5 ml of 0.75% ropivacaine was perpendicularly injected into the subcutaneous space at this point. The needle was redirected along this plane superiorly and inferiorly. After test aspiration, 5 ml of 0.75% ropivacaine was injected into the subcutaneous space. This technique was performed bilaterally.
5. After induction in both the G1 and G2 patients, 40 mg intravenous pantoprazole, 2 g cefazolin, 30 mg ketorolac, 8 mg ondansetron, and 10 mg dexamethasone were administered. At the end of the surgery, the patients were extubated in a delicate manner that avoided coughing and were then transferred to the postanesthesia care unit (PACU).

### Thyroidectomy

All enrolled patients were submitted to total thyroidectomy by an anterior cervicotomy, utilizing the conventional technique and without the use of any hemostatic instrument. None of the patients underwent cervical drainage.

### Procedures in the PACU

The prescription for the G1 and G2 patients was as follows: 5 L/minute oxygen nebulization, 30 degrees of head elevation, and 0.15 mg/kg nalbuphine, as needed for pain. Pain was evaluated by a visual analog scale (VAS) that ranged from 0 to 10, with a score of 0 indicating no pain; a score of 1 to 3 indicating mild pain; a score of 4 to 7 indicating moderate pain; and a score of 8 to 10 indicating intense pain. If the VAS score was above 3, then medication was administered. These doses were repeated every 20 minutes as necessary. Each patient remained in the PACU for 60 minutes. During this period, the following data were recorded every 15 minutes:

1. Vital signs: noninvasive blood pressure (NIBP), HR, pulse oximetry, axillary temperature, and glycemia (if the patient has been previously diagnosed with diabetes);
2. Pain by the VAS;
3. The presence or absence of side effects, including shivering, agitation, dyspnea, nausea and vomiting, cervical hematoma, and bleeding. Postoperative nausea and vomiting (PONV) were evaluated using the PONV scale 5, with a score of 1 indicating no nausea; 2 indicating mild nausea; 3 indicating severe nausea; and 4 indicating vomiting. For scores of 3 and 4, an antiemetic drug was administered.

### Procedures in the Inpatient Unit (IU)

The prescription for the G1 and G2 patients was as follows: soft meal, 40 mg intravenous (i.v.) pantoprazole once per day, 30 mg i.v. ketorolac (8/8 hours), and 0.2 mg/kg i.v. nalbuphine (6/6 hours) for pain (VAS>3); the patients were additionally administered 8 mg ondansetron if the PONV score was 3 or 4.

Vital signs, including NIPB, HR, and pulse oximetry, and the occurrence of side effects (nausea and vomiting, shivering, agitation, dyspnea, and cervical hematoma) were evaluated every 4 hours.

### Statistical Analyses

Categorical variables are presented in tables as absolute (n) and relative (%) frequencies. The association between groups was evaluated by the chi-square test, Fisher's exact test, or the likelihood ratio test. The quantitative variables were presented as the mean and standard deviation and were defined as parametric by the Kolmogorov-Smirnov test. Repeated measures ANOVA (rm-ANOVA), with Bonferroni's post hoc test when necessary, was applied for comparisons between groups of continuous variables; Student's t-test was also used to compare two independent groups of quantitative data. *p*-values <0.05 were considered statistically significant.

## RESULTS

Among the 100 participants in this study, 18% were men and 82% were women. The average patient age in G1 was 45 years old, and the average patient age in G2 was 50 years old. No significant difference was found in ASA physical status between the groups. The most prevalent comorbidities in both groups were diabetes (6% in G1 and 14% in G2), systemic arterial hypertension (28% in G1 and 26% in G2), and dyslipidemia (12% in G2); however, with the exception of dyslipidemia, there were no significant differences between these rates. The main surgical diagnoses were goiter (62% in both G1 and G2), differentiated carcinoma (38% in G1 and 36% in G2), and toxic goiter (2% in G2); there were no significant differences between the groups. In G1, there were two cases of difficult airways, and intubations with bronchoscopy were performed by the anesthesiologist. Descriptive data and the results of homogeneity analysis are described in Table 1.

### Data from the OR

The average surgical time was 120 minutes (range: 104-140 minutes), and the average anesthesia time was 135 minutes (range: 120-150 minutes). There was no significant difference between the groups (*p*=0.682, Student's t-test). The NIABP, HR and temperature values at the three time points were similar and had the same behavior over time for the G1 and G2 patients during the surgery. The comparison between groups for the OR variables and descriptive data are described in Table 2.

### Data from the PACU

Upon arrival in the PACU, there was no significant difference between the NIABP in the two groups. However, over time (at 15, 30, 45, and 60 minutes), significant differences were observed between the two groups. The main NIABP was significantly lower in G2 patients than in G1 patients (*p*<0.001; rm-ANOVA). The HR was significantly lower in G2 patients than in G1 patients at PACU arrival and 15, 30, 45, and 60 minutes after surgery (*p*<0.001; rm-ANOVA and Bonferroni's post hoc test). In the PACU, the temperature data obtained for the two groups were the same, without significant differences.

There were significant differences (*p*<0.001; Fisher's exact test) in the VAS scores at 15 and 30 minutes after arrival in the PACU. After 15 minutes, 56% of G1 patients reported pain (VAS>3), compared to only 6% of G2 patients. A dose of nalbuphine was administered. After 30 minutes, 34% of G1 patients and only 2% of G2 patients reported pain, and another dose of nalbuphine was necessary. After 45 minutes, 16% of G1 patients reported pain, while none of the patients in G2 reported pain (*p*=0.006; Fisher's exact test). After 60 minutes, there were no significant differences between the groups.

Opioid consumption was significantly lower in G2 patients than in G1 patients at 15 and 30 minutes upon arrival in the PACU (*p*<0.001 for both analyses; Fisher's exact test). After 15 minutes, 56% of G1 patients required the administration of nalbuphine compared to only 6% of G2 patients. After 30 minutes, 34% of G1 patients required an additional dose compared to only 2% of G2 patients. At 45 minutes, 16% of G1 patients required another dose of medication compared to 0% of G2 patients (*p*=0.006; Fisher's exact test). After 60 minutes, there was no significant difference.

In the PACU at 45 and 60 minutes, 16% of the patients in G1 had nausea and vomiting (PONV 3 or 4) compared to 0% of G2 patients (*p*=0.006 for both analyses; Fisher's exact test).

In G1, there was 1 case of cervical hematoma in the first 60 minutes (2%). No cases of hematoma were observed in G2. However, this was not a significant difference. The single case was not treated surgically because it was a small, stable subcutaneous hematoma. In the PACU, there were no cases of dyspnea in either group.

The complete analyses of both groups in the PACU are described in Table 3.

### Data from the IU

There were no significant differences between the main NIABP, HR, or temperature between the G1 and G2 patients in the IU.

At 4 hours after arrival, 20% of G1 patients reported pain (VAS>3), and an additional opioid dose was necessary. In G2, only 6% of patients reported pain (*p*=0.037; Fisher's exact test). At the other time points (after 4 hours), there was no significant difference.

Opioid consumption between the groups had the same behavior as the pain scores. In G1, 4 hours after arrival, 20% of patients requested nalbuphine, while only 6% of patients in G2 requested nalbuphine (*p*=0.037; Fisher's exact test). After 8 and 12 hours, there were no differences in opioid consumption between the groups.

G1 patients had significantly more nausea and vomiting (PONV 3 or 4) than G2 patients during the postoperative period. In G1, 18% and 14% of patients reported PONV at 4 and 8 hours, respectively, compared to 0% of patients in G2 (*p*=0.003 and *p*=0.012, respectively; Fisher's exact test).

There was no significant difference in the incidence of hematoma in the IU. Upon arrival, only 1 case of hematoma had occurred, corresponding to the same case in the PACU (2%). After 4 hours, another case of hematoma occurred in G1. After 12 hours, the incidence of hematoma was 4% in G1 and 0% in G2. Neither case required surgical re-exploration because both were small subcutaneous hematomas. There were no cases of dyspnea or shivering in either group.

The complete analysis of the IU period is shown in Table 4.

## DISCUSSION

Recent studies have correlated pain, thyroidectomy, and BSCPB 5-10, but the efficacy of BSCPB for the postoperative period remains controversial. The present study demonstrated that patients undergoing BSCPB in combination with general anesthesia had increased pain control, reduced opioid consumption, and a reduced incidence of nausea and vomiting.

First, it is important to note that this study was a prospective study and that the period of observation was during the hospital stay, and data acquisition was performed by a single team. Moreover, there were no dropout or missing patients in the study, and only patients with ASA I (healthy) or ASA II (systemic disease compensated) were included. Patients with rheumatic or neurological diseases (central or peripheral) and patients being treated for chronic pain or other conditions were excluded.

Upon PACU arrival, almost no patients reported pain in either group, possibly due to the residual effects of the anesthesia administered in the OR. However, the VAS scores and opioid consumption (nalbuphine) were significantly lower for PACU and IU patients than for BSCPB patients. Gürkan et al. 9 first demonstrated an association between BSCPB and a reduction in opioid consumption. Opioid consumption was related to pain scores reported by patients in the present study. If the VAS score was >3, one dose was administered after each evaluation. It is also possible to correlate the lower pain scores reported by G2 patients with the hemodynamic parameters in the PACU. The NIABP and HR of the patients who received the blockade were 12% lower than those of the patients who received general anesthesia, and opioid consumption reduction was 70%, probably because the adrenergic response caused by postoperative stress (pain) was less intense.

Sardar et al. 11 demonstrated no differences between groups under general anesthesia and groups under general anesthesia combined with BSCPB. However, the authors used just 15 ml 0.25% bupivacaine, and the anesthesia gradually becomes less potent with the gradual reduction in local anesthetic concentration; this method does not reflect effective pain control at the PACU and IU. Based on that, the difference in pain control found in the present study in patients treated with BSCPB could be justified because we used ropivacaine at a concentration of 0.75%, which leads to a longer lasting anesthetic effect.

Mukhopadhyay et al. 12 demonstrated no advantage of BSCPB for thyroid surgeries. In that study, patients underwent blockade with 0.5% ropivacaine. However, the technique applied included two punctures with a total volume of 6 ml on each side. In a blockade, it is important that the anesthetic is spread to as many fibers as possible. The spread of anesthetic is proportional to its volume. In the present study, 10 ml of ropivacaine was administered on each side. Nevertheless, Mukhopadhyay et al. 12 compared the blockade, the sole anesthetic technique, with general anesthesia. In our study, all patients received general anesthesia. The blockade was added to compare the postoperative conditions and patient outcomes. Moreover, no blockade complications were observed.

The volume and concentration of the anesthetic are important for local anesthetic efficacy 13. Herbland et al. 14 demonstrated no efficacy of the blockade with 0.75% ropivacaine in relieving postoperative pain for 36 hours after the operation. The present study evaluated only the first 12 postoperative hours.

Cai et al. 7 demonstrated an association between BSCPB and a reduction in PONV, and this association corroborates the findings of the present study. Nausea and vomiting appeared late in the PACU and was more frequent in those patients not submitted to BSCPB. G2 patients reported lower pain scores (VAS<3) over time, consumed fewer opioids, and presented with fewer side effects, such as nausea and vomiting, than G1 patients. This observation may occur because PONV is likely a side effect of opioid administration; furthermore, these attributes are cumulative and time- and dose-dependent.

More recent studies have demonstrated the efficacy of the blockade for thyroidectomies. Kannan et al. 15 demonstrated a reduced use of sevoflurane during surgery, and Awekes et al. 16 demonstrated a reduced use of perioperative opioids, namely, during the surgery and the postoperative period.

However, a relevant aspect of the present study must be considered. The anesthetic technique used during the procedure in both groups was TIVA. With this technique, anesthesia is induced with a single dose of fentanyl (2 mcg/kg) and maintained with a continuous infusion of propofol and remifentanil. As a result, there is no residual effect of analgesia in the recovery room. All the previously mentioned studies used sevoflurane (balanced anesthesia technique) or repeated fentanyl doses for the maintenance of anesthesia during the surgery. Sevoflurane maintenance and additional doses of fentanyl provide residual analgesic effects in the postoperative period, mainly in the recovery room. It is possible that the analgesic effects demonstrated by previous studies were not the unique effect of the cervical plexus blockade but were the additional residual effects of the opioids and inhaled drugs from the anesthetic technique applied in the OR.

Mayhew et al. 17 demonstrated the analgesic efficacy of the blockade in a meta-analysis. However, the general anesthesia technique used was not mentioned or compared. This important aspect may influence analgesia during the earlier postoperative period. The meta-analysis did not find a reduction in PONV, contrary to the results of the present study. PONV is related to the amount of opioids used (side effects) and to the anesthesia technique used 18.

Senapathi et al. 19 and Gürkan et al. 9 compared the efficacy of the blockade between the standard and the ultrasound-guided technique. In fact, the ultrasound-guided technique is an additional step that will be tested after the conclusion of this study.

The present study has some important limitations. Notably, no sample size estimation was performed a priori. In fact, authors designed the study to be one of the largest in this specific literature category. Additionally, other limitations may include problems with concealment and blinding procedures, the absence of a placebo control group, the simple randomization method and the risk of imbalance between groups.

In conclusion, the present study demonstrated that BSCPB using the landmark technique associated with TIVA for thyroidectomy is feasible, safe, and effective for improving patient outcomes based on pain control, reduced opioid consumption during the first 4 postoperative hours, and reduced nausea and vomiting during the first 8 hours of the postoperative period.

## AUTHOR CONTRIBUTIONS

Araujo-Filho VJF and Gourlart TF were responsible for the study design. Goulart TF and Matos LL were responsible for the data acquisition. Cernea CR was responsible for the manuscript conception and review. All of the authors read and approved the final version of the manuscript.

## Figures and Tables

**Table 1:** Descriptive data and homogeneity analysis.

Variable	Group 1	Group 2	*p*-value (test)
Male sex, n (%)	9 (18%)	9 (18%)	1.000 (Pearson's chi-square)
Age, mean ± SD, years	50±15	46±13	0.119 (Student's t-test)
BMI, mean ± SD, kg/m^2^	26±4	27±5	0.361 (Student's t-test)
ASA, n (%)			
I	27 (54%)	21 (42%)	0.230 (Pearson's chi-square)
II	23 (46%)	29 (58%)	
Comorbidities, n (%)			
Systemic arterial hypertension	14 (28%)	13 (26%)	0.822 (Pearson's chi-square)
Dyslipidemia	0 (0%)	6 (12%)	0.012 (Fisher's exact test)
Diabetes	3 (6%)	7 (14%)	0.182 (Fisher's exact test)
Obesity	3 (6%)	5 (10%)	0.715 (Fisher's exact test)
Surgical diagnosis, n (%)			
Nodular goiter	31 (62%)	31 (62%)	
Toxic goiter	0 (0%)	1 (2%)	0.493 (likelihood ratio)
Differentiated carcinoma	19 (38%)	18 (36%)	
Difficult airway and intubation with bronchoscopy, n (%)	0 (0%)	2 (4%)	0.495 (Fisher's exact test)

Legend: SD, standard deviation; BMI, body mass index.

**Table 2:** Noninvasive arterial blood pressure (NIABP), heart rate (HR), and temperature in the operating room (OR).

Variable	Group 1	Group 2	*p*-value (test)
NIABP, mean ± SD, mmHg			
M1	82.9±10.8	85.1±10.1	
M2	65.2±12.5	68.8±12.6	0.562 (rm-ANOVA)
M3	83.6±11.6	86.2±12.5	
HR, mean ± SD, bpm			
M1	63.6±9.9	66.4±8.1	
M2	62.9±9.9	65.2±11.1	0.756 (rm-ANOVA)
M3	71.8±9.8	74.3±10.3	
Temperature, mean ± SD, °C			
M1	36.2±0.2	36.3±0.2	
M2	36.2±0.2	36.2±0.3	0.083 (rm-ANOVA)
M3	36.2±0.2	36.0±0.3	

Legend: SD, standard deviation; rm-ANOVA, repeated measures ANOVA.

**Table 3:** Noninvasive arterial blood pressure (NIABP), heart rate (HR), temperature, pain scores (VAS>3), opioid (nalbuphine) consumption, postoperative nausea and vomiting (PONV), and cervical hematoma in the postanesthesia care unit (PACU).

Variable	Group 1	Group 2	*p*-value (test)
NIABP, mean ± SD, mmHg			<0.001 (rm-ANOVA)
Arrival	88.7±13.0	88.1±13.7	0.856 (Bonferroni)
15 minutes	100.7±14.6	90.2±10.8	<0.001 (Bonferroni)
30 minutes	103.8±11.3	92.2±10.7	<0.001 (Bonferroni)
45 minutes	101.1±10.5	91.6±11.9	<0.001 (Bonferroni)
60 minutes	101.0±11.1	92.3±10.4	<0.001 (Bonferroni)
HR, mean ± SD, bpm			<0.001 (rm-ANOVA)
Arrival	87.4±9.2	74.2±6.0	<0.001 (Bonferroni)
15 minutes	87.8±8.9	75.3±6.1	<0.001 (Bonferroni)
30 minutes	88.4±8.4	77.2±7.1	<0.001 (Bonferroni)
45 minutes	87.9±9.3	77.5±7.4	<0.001 (Bonferroni)
60 minutes	87.9±8.9	78.0±6.8	<0.001 (Bonferroni)
Temperature, mean ± SD, °C			0.314 (rm-ANOVA)
Arrival	36.0±0.3	35.9±0.4	-
15 minutes	36.0±0.3	35.9±0.3	-
30 minutes	36.1±0.2	36.0±0.3	-
45 minutes	36.2±0.2	36.1±0.2	-
60 minutes	36.3±0.2	36.1±0.2	-
Pain scores (VAS>3), n (%)			
Arrival	10 (20%)	4 (8%)	0.084 (Fisher's exact test)
15 minutes	28 (56%)	3 (6%)	<0.001 (Fisher's exact test)
30 minutes	17 (34%)	1 (2%)	<0.001 (Fisher's exact test)
45 minutes	8 (16%)	0	0.006 (Fisher's exact test)
60 minutes	4 (8%)	0	0.117 (Fisher's exact test)
Opioid (nalbuphine) consumption, n (%)			
Arrival	10 (20%)	4 (8%)	0.084 (Fisher's exact test)
15 minutes	28 (56%)	3 (6%)	<0.001 (Fisher's exact test)
30 minutes	17 (34%)	1 (2%)	<0.001 (Fisher's exact test)
45 minutes	8 (16%)	0	0.006 (Fisher's exact test)
60 minutes	4 (8%)	0	0.117 (Fisher's exact test)
PONV, n (%)			
Arrival	0	2 (4%)	0.495 (Fisher's exact test)
15 minutes	1 (2%)	1 (2%)	1.000 (Fisher's exact test)
30 minutes	5 (10%)	2 (4%)	0.436 (Fisher's exact test)
45 minutes	8 (16%)	0	0.006 (Fisher's exact test)
60 minutes	8 (16%)	0	0.006 (Fisher's exact test)
Cervical hematoma, n (%)			
Arrival	0	0	-
15 minutes	0	0	-
30 minutes	0	0	-
45 minutes	1 (2%)	0	1.000 (Fisher's exact test)
60 minutes	1 (2%)	0	1.000 (Fisher's exact test)

Legend: SD, standard deviation; rm-ANOVA, repeated measures ANOVA; Bonferroni, Bonferroni's post hoc test.

**Table 4:** Noninvasive arterial blood pressure (NIABP), heart rate (HR), temperature, pain scores (VAS>3), opioid (nalbuphine) consumption, postoperative nausea and vomiting (PONV), and cervical hematoma in the inpatient unit (IU).

Variable	Group 1	Group 2	*p*-value (test)
NIABP, mean ± SD, mmHg			0.465 rm-ANOVA
Arrival	91.0±24.8	90.4±9.0	-
4 hours	91.6±9.6	90.4±15.1	-
8 hours	94.5±14.0	87.2±9.1	-
12 hours	92.4±9.1	86.8±12.8	-
HR, mean ± SD, bpm			0.115 rm-ANOVA
Arrival	79.5±10.5	80.3±10.0	-
4 hours	76.1±9.2	78.4±10.4	-
8 hours	76.9±8.5	77.5±9.4	-
12 hours	78.7±8.3	76.7±8.0	-
Temperature, mean ± SD, °C			0.237 rm-ANOVA
Arrival	36.2±0.3	36.1±0.3	-
4 hours	36.3±0.3	36.1±0.4	-
8 hours	36.3±0.3	36.2±0.4	-
12 hours	36.3±0.3	36.3±0.4	-
Pain scores (VAS>3), n (%)			
Arrival	6 (12%)	5 (10%)	0.749 (Fisher's exact test)
4 hours	10 (20%)	3 (6%)	0.037 (Fisher's exact test)
8 hours	5 (10%)	3 (6%)	0.715 (Fisher's exact test)
12 hours	4 (8%)	1 (2%)	0.362 (Fisher's exact test)
Opioid (nalbuphine) consumption, n (%)			
Arrival	6 (12%)	5 (10%)	0.749 (Fisher's exact test)
4 hours	10 (20%)	3 (6%)	0.037 (Fisher's exact test)
8 hours	5 (10%)	3 (6%)	0.715 (Fisher's exact test)
12 hours	4 (8%)	1 (2%)	0.678 (Fisher's exact test)
PONV, n (%)			
Arrival	2 (4%)	0	0.495 (Fisher's exact test)
4 hours	9 (18%)	0	0.003 (Fisher's exact test)
8 hours	7 (14%)	0	0.012 (Fisher's exact test)
12 hours	6 (12%)	2 (4%)	0.269 (Fisher's exact test)
Cervical hematoma, n (%)			
Arrival	1 (2%)	0	1.000 (Fisher's exact test)
4 hours	2 (4%)	0	0.495 (Fisher's exact test)
8 hours	2 (4%)	0	0.495 (Fisher's exact test)
12 hours	2 (4%)	0	0.495 (Fisher's exact test)

Legend: SD, standard deviation; rm-ANOVA, repeated measures ANOVA; Bonferroni, Bonferroni's post hoc test.
